# Supplementary material for: Tooth position prediction method based on adaptive geometry optimization
Source: PLoS One. 2025 Jul 7;20(7):e0327498. doi: 10.1371/journal.pone.0327498 (PMC12233242; doi:10.1371/journal.pone.0327498)
Supplement: Attachment — Submitted filename: Rebuttal_Letter.pdf [file pone.0327498.s001.pdf]

Answer all comments of the reviewers：

1. The introduction effectively presents the importance of the research problem, but it could benefit from a more concise overview of the existing methods and their limitations. Consider streamlining the discussion of prior works to avoid redundancy and emphasize the gap this paper addresses more explicitly.

Answer: Dear Expert, I have simplified the introduction section, with the red-highlighted portions reflecting the revised content. The main change involves streamlining the discussion of previous work, and reducing it by approximately half.

2. The abstract is informative but overly detailed. Simplify technical jargon where possible, focusing on the core contributions and results. For instance, the introduction of specific methods such as GAOS and PARS could be summarized without extensive technical detail to improve readability.

Answer: Dear Expert, I have simplified the technical terms related to GAOS and PARS in the red-highlighted sections of the abstract to make it more focused on the core contributions and results. The main revisions are located in lines 4 to 7 of the abstract.

3. The transition between sections is occasionally abrupt, particularly between the "Related Work" and "Methodology" sections. Adding bridging sentences to explain how the literature review informs the methodology would improve the coherence and flow.

Answer: Dear Expert, I have added bridging sentences in lines 1 to 14 of the third paragraph of the related work section to improve the transition. The changes have been highlighted in red.

4. Consider providing more detailed justifications for the choice of the physiological adaptive reconstruction strategy. While the use of physiological characteristics is mentioned, the connection to clinical relevance and practical orthodontic application could be more clearly articulated.

Answer: Dear Expert, I have added the clinical relevance and connection to practical orthodontic applications for the Physiological Adaptive Reconstruction Strategy. The main revisions can be found in lines 5 to 11 of the first paragraph, highlighted in red.

5. The manuscript lacks a clear explanation of the limitations and potential drawbacks of the proposed methods. It is crucial to acknowledge any challenges or potential issues to provide a balanced perspective and inform future research directions.

Answer: Dear Expert, I have added an explanation of the limitations, potential drawbacks and the scope for further optimization of the proposed method in the

conclusion section. The revisions can be found in lines 11 to 17 of the conclusion section, it has highlighted in red.

6. The figures, particularly those showing algorithmic flow (e.g., Figs 1 and 2), could be improved by adding clearer captions that explain their relevance in the context of the methodology. Captions should be more descriptive to guide readers unfamiliar with the concepts presented.

Answer: Dear Expert, I have revised the titles of the figures for better clarity. The title of Fig 1 has been changed from "GAOS Diagram" to "Effect of GAOS Strategy to Generate Dataset" and the title of Fig 2 has been changed from "PARS Diagram" to "Effect of PARS Strategy to Generate Dataset" in order to better reflect their functions and concepts for easier understanding by the readers.

7. The mathematical formulations provided in the methodology are detailed, but there are occasional inconsistencies in notation and terminology. Ensure uniformity in the description of symbols across all equations, and clarify the definitions where symbols are first introduced.

Answer: Dear Expert, after revisiting the details of the mathematical formulas, I have corrected the consistency in the symbol descriptions and provided explanations for symbols introduced for the first time. The main revisions are in Eq(4) – (6), (8) – (9) and (12) – (16), with the changes have highlighted in red.

8. Some sections are dense with technical language, which may hinder comprehension for a broader audience. For example, the physiological adaptive reconstruction strategy's description could benefit from simplified language or a supplementary explanation targeted at non-specialist readers.

Answer: Dear Expert, I have simplified the technical language of the Physiological Adaptive Reconstruction Strategy. The main revisions are in lines 1 to 4 of the first paragraph, and it has highlighted in red to facilitate easier reading for non-specialist readers.

9. The experimental results are promising, but the discussion section would benefit from a more thorough comparative analysis with state-of-the-art methods. Specifically, providing statistical significance testing of the improvements reported would add rigor and strengthen the validity of the findings.

Answer: Dear Expert, the method is compared with the physiological dynamic optimization strategy in this study is the one published in 2020 in the International Conference on Medical Image Computing and Computer-Assisted Intervention. This paper is currently the only one in the field of tooth position prediction that proposes

an optimization strategy specifically for datasets, which is why I selected it as the comparison method.
The methods compared with the F-VAMP network are published in 2022 on the IEEE Transactions on Visualization and Computer Graphics and in 2020 on the European Conference on Computer Vision. These papers are of high academic quality in the field of tooth position prediction and are published recently. Additionally, most of the recent literature on orthodontics focuses on tooth arrangement rather than tooth position prediction. Therefore, this study selects these papers as comparison methods for the experimental analysis of tooth position prediction.
Furthermore, the statistical significance metrics were obtained by training and testing datasets derived from 834 original data sets, which were split in an 8:2 ratio, using the GAOS and PARS strategies. I have conducted a more thorough comparative analysis of the methods being compared, including the innovative technical details of the GAOS and PARS strategies, as well as a comparative evaluation of these strategies.
On the other hand, the comparison analysis of F-VAMP provides a more in-depth evaluation of both the shortcomings of the comparison methods and the advantages of the proposed method. The revisions are highlighted in red in the sections Comparison and Ablation of Physiological Dynamic Optimization Strategy and Comparison and Ablation of F-VAMP Networks.

10. The conclusion is well-structured but could be expanded to include a more explicit discussion of potential applications in clinical settings and future research opportunities that could further extend the work presented here.

Answer: Dear Expert, I have made revisions in the Conclusion section, specifically in lines 1 to 10 and 15 to 17, where the red-highlighted text now provides a clearer discussion of the current research context and future research opportunities. Additionally, I have further elaborated on the contributions of the proposed method.

Answer all Additional Editor Comments:

1. The introduction could benefit from a more detailed review of existing literature to better contextualize the novelty of the proposed method.

Answer: Dear Expert, I have provided a more detailed review of the existing literature, with specific revisions highlighted in red. These changes can be found in the second paragraph of the related work section, in lines 2 to 11 and 17 to 23.

2. The methods could be enhanced by providing more information on the dataset used for training and testing, including the size and diversity of the data. Details on the computational resources and time required for training the model would be beneficial.

Answer: Dear Expert, I have provided additional information on the size and diversity of the training and testing datasets to strengthen these methods, along with a detailed description of the experimental setup. The specific details can be found in the Dataset and Implementation Details section and are highlighted in red.

3. The results section could be strengthened by including more visualizations of the predicted tooth positions compared to the ground truth.

Answer: The statistical metrics include the quantified translations and rotations of the predicted positions. Additionally, two sets of visualizations illustrating the experimental effects have been added to Fig. 7. Due to space limitations in Fig. 7, the quantified data regarding the predicted positions and ground truth are provided in Table 3.

4. Statistical significance of the improvements should be discussed to reinforce the validity of the results.

Answer: Dear Expert, The statistical significance metrics were obtained by training and testing datasets derived from 834 original data sets, split in an 8:2 ratio, using the GAOS and PARS strategies. Additionally, I have conducted a more thorough comparative analysis of the methods being compared, with a particular focus on the innovative technical details of the GAOS and PARS strategies, as well as a detailed comparison between the two.
Furthermore, the comparison analysis of F-VAMP provides a more in-depth evaluation of both the shortcomings of the comparison methods and the advantages of the proposed method. The revisions are highlighted in red.

5. The discussion could benefit from a more critical analysis of the limitations of the study and potential areas for future research. The impact of the proposed method on clinical workflows and patient outcomes could be elaborated.

Answer: Dear Expert, I have added an explanation of the limitations, potential drawbacks, and the scope for further optimization in future research regarding the proposed method in the Conclusion section. The revisions can be found in lines 11 to 17 of the Conclusion, highlighted in red.

6. The conclusion could be more forward-looking by suggesting specific future research directions or potential applications of the method in other areas of dental research.

Answer: Dear Expert, I have made revisions in the Conclusion section, specifically in lines 1 to 10 and 15 to 17, where the red-highlighted text now offers a clearer discussion of the current research context and future research opportunities. Additionally, I have further elaborated on the contributions of the proposed method.

7. Overall, the manuscript could be improved by providing more detailed information on the dataset, computational resources, and statistical significance of the results.A more comprehensive review of related work and a critical discussion of limitations would enhance the manuscript's depth.

Answer: Dear Expert, I have provided the code and sample dataset for this study at the following URL: https://gitcode.com/zyj_jj/TPPMAGO. All datas are stored in URL: https://gitcode.com/zyj_jj/TPPMAGO_toothdata/blob/main/README.md
Improvements have been made to the statistical significance of the physiological dynamic optimization strategy and the F-VAMP network, with a more thorough analysis from multiple perspectives. More detailed information on the dataset and computation methods is provided in the Dataset and Implementation Details section.
Additionally, I have conducted a more comprehensive review of the related work and provided a critical discussion of the limitations. The specific revisions are highlighted in red in the second paragraph of the Related Work section, in lines 2 to 11 and 17 to 23.

All Journal Requirements:

1. Please ensure that your manuscript meets PLOS ONE's style requirements, including those for file naming.

Answer: Dear Editor, this manuscript has been written strictly according to the latest LaTeX template provided on the PLOS ONE website, and the formatting has been adjusted in accordance with the submission guidelines specified on the PLOS ONE website.

2. Please note that PLOS ONE has specific guidelines on code sharing for submissions in which author-generated code underpins the findings in the manuscript. In these cases, we expect all author-generated code to be made available without restrictions upon publication of the work. Please review our guidelines at https://journals.plos.org/plosone/s/materials-and-software-sharing#loc-sharing-code and ensure that your code is shared in a way that follows best practice and facilitates reproducibility and reuse.

Answer: Dear Editor, the code for this manuscript can be accessed at the following URL: https://gitcode.com/zyj_jj/TPPMAGO, where the dataset folder contains the sample dataset. All the datasets are stored in the following link: https://gitcode.com/zyj_jj/TPPMAGO_toothdata/blob/main/README.md

3. We note that the grant information you provided in the 'Funding Information' and 'Financial Disclosure' sections do not match.

Answer: Dear expert, I have uploaded the latest Financial Disclosure Statement report as "Other" in the online submission of the Attach Files system. To ensure that the "Funding Information" matches the "Financial Disclosure".

4. We note that you have indicated that there are restrictions to data sharing for this study. PLOS only allows data to be available upon request if there are legal or ethical restrictions on sharing data publicly. For more information on unacceptable data access restrictions, please see http://journals.plos.org/plosone/s/data-availability#loc-unacceptable-data-access-restrictions.

Before we proceed with your manuscript, please address the following prompts:

a) If there are ethical or legal restrictions on sharing a de-identified data set, please explain them in detail (e.g., data contain potentially identifying or sensitive patient information, data are owned by a third-party organization, etc.) and who has imposed them (e.g., a Research Ethics Committee or Institutional Review Board, etc.). Please also provide contact information for a data access committee, ethics committee, or other institutional body to which data requests may be sent.

b) If there are no restrictions, please upload the minimal anonymized data set necessary to replicate your study findings to a stable, public repository and provide us with the relevant URLs, DOIs, or accession numbers. For a list of recommended repositories, please see https://journals.plos.org/plosone/s/recommended-repositories. You also have the option of uploading the data as Supporting Information files, but we would recommend depositing data directly to a data repository if possible.

Answer: Dear Editor, I have made the code publicly available, and the corresponding URL is: https://gitcode.com/zyj_jj/TPPMAGO, where the dataset folder contains the sample dataset. All datas are stored in URL: https://gitcode.com/zyj_jj/TPPMAGO_toothdata/blob/main/README.md

Note: Dear Editor, due to a change in the name of my institution in April 2025, I have updated the name of the college from the former "College of Computer Science and Technology" to the new "College of Artificial Intelligence & Computer Science."
