# Supplementary material for: Tooth position prediction method based on adaptive geometry optimization
Source: PLoS One. 2025 Jul 7;20(7):e0327498. doi: 10.1371/journal.pone.0327498 (PMC12233242; doi:10.1371/journal.pone.0327498)
Supplement: Attachment — Submitted filename: Response to Reviewers.pdf [file pone.0327498.s002.pdf]

Answer all comments of the reviewers：

1. The English writing of the manuscript needs to be significantly improved. There are many typos in the current manuscript. For example, in lines 93-94, two sentences are repeated. Grammar mistakes should be corrected to make the manuscript readable.

Answer: Regarding the issue, we have removed the redundant sentence in lines 93-94. Furthermore, we have checked the entire manuscript, corrected all the typos errors and improved the English writing. These modifications are specifically reflected in red, such as lines 103-105, 119, 176-177, 192-200, 229, 239, 386, 419, 426-435, 446, and 450-452, and so on.

2. Many repeated details of the proposed two strategies and transformer architecture should be omitted to make expressions clear and easy to read.

Answer: I have omitted many of the repetitive details regarding the proposed two strategies and the Transformer. The modifications to the GAOS strategy are reflected in lines 124-131, the changes to the PARS strategy are reflected in lines 149-156, and the adjustments to the Transformer architecture are shown in lines 210-221. The specific changes have been highlighted in red text.

3. The presentation of the proposed method and experimental results should be improved. For example, authors can enrich the captions and illustrations under each figure.

Answer: The content of Figures 1 to 8 has been enriched in this paper. The specific modifications are located after the titles of Figures 1 to 8(between lines 131-132, 168-169, 226-227, 243-244, 267-268, 287-288, and 424-425), and the changes have been highlighted in red text.

All Journal Requirements:

Please review your reference list to ensure that it is complete and correct. If you have cited papers that have been retracted, please include the rationale for doing so in the manuscript text, or remove these references and replace them with relevant current references. Any changes to the reference list should be mentioned in the rebuttal letter that accompanies your revised manuscript. If you need to cite a retracted article, indicate the article's retracted status in the References list and also include a citation and full reference for the retraction notice.

Answer: I have checked the status of each reference cited in this paper through Google Scholar, and all of them are published, complete and correct references.
